# Organizational capacities of national pharmacovigilance centres in Africa: assessment of resource elements associated with successful and unsuccessful pharmacovigilance experiences

**DOI:** 10.1186/s12992-018-0431-0

**Published:** 2018-11-16

**Authors:** H. Hilda Ampadu, Jarno Hoekman, Daniel Arhinful, Marilyn Amoama-Dapaah, Hubert G. M. Leufkens, Alex N. O. Dodoo

**Affiliations:** 1 The African Collaborating Centre for Pharmacovigilance, 1 Vigilance Place, Mango Tree Avenue, Asylum Down, Accra, Ghana; 2 WHO Collaborating Centre for Pharmaceutical Policy and Regulation, Division of Pharmacoepidemiology & Clinical Pharmacology, Utrecht Institute for Pharmaceutical Sciences (UIPS), Utrecht University, 3508 TB Utrecht, the Netherlands; 3 Innovation Studies Group, Copernicus Institute for Sustainable Development, Faculty of Geosciences, Utrecht University, 3508 TB Utrecht, the Netherlands; 4 Noguchi Memorial Institute for Medical Research, University of Ghana, P. O Box LG 25, Accra, Ghana

**Keywords:** National pharmacovigilance centres, Organizational capacity, Resource elements, Stakeholders, Outcomes, National governments, Development partners, Public health Programmes

## Abstract

**Background:**

National pharmacovigilance centres (national centres) are gradually gaining visibility as part of the healthcare delivery system in Africa. As does happen in high-income countries, it is assumed that national centres can play a central coordinating role in their national pharmacovigilance (PV) systems. However, there are no studies that have investigated whether national centres in Africa have sufficient organizational capacity to deliver on this mandate and previous studies have reported challenges such as lack of funding, political will and adequate human resources.

We conducted interviews with strategic leaders in national centres in 18 African countries, to examine how they link the capacity of their organization to the outcomes of activities coordinated by their centres. Strategic leaders were asked to describe three situations in which activities conducted by their centre were deemed successful and unsuccessful. We analyzed these experiences for common themes and examined whether strategic leaders attributed particular types of resources and relationships with stakeholders to successful or unsuccessful activities.

**Results:**

We found that strategic leaders most often attributed successful experiences to the acquisition of political (e.g. legal mandate) or technical (e.g. active surveillance database) resources, while unsuccessful experiences were often attributed to the lack of financial and human resources. Stakeholders that were most often mentioned in association with successful experiences were national government and development partners, whereas national government and public health programmes (PHPs) were often mentioned in unsuccessful experiences. All 18 centres, regardless of maturity of their PV systems had similar challenges.

**Conclusions:**

The study concludes that national centres in Africa are faced with 3 core challenges: (1) over-reliance on development partners, (2) seeming indifference of national governments to provide support after national centres have gained membership of the World Health Organization (WHO) Programme for International Drug Monitoring (PIDM) and (3) engaging public health programmes in a sustainable way.

**Electronic supplementary material:**

The online version of this article (10.1186/s12992-018-0431-0) contains supplementary material, which is available to authorized users.

## Background

The last years have witnessed increasing efforts in low and middle income countries to establish formal national pharmacovigilance centres (national centres) with several of these in sub-Saharan Africa [1, 2]. Pharmacovigilance (PV) became an important discipline in the 1960s following the thalidomide tragedy [3]. The realization that the tragedy could have been prevented if countries collected and shared data on medicine safety led the World Health Organization (WHO) decision-making body i.e. the World Health Assembly to issue a resolution inviting “Member States to arrange for a systematic collection of information on serious adverse drug reactions (ADR) observed during the development of a drug and, in particular, after its release for general use” ([4], pp 14). In response to this call, national governments around the world established national pharmacovigilance centres to coordinate medicine safety surveillance efforts. Over the years these centres have become key organizations involved in initiating, building and sustaining efforts for safety surveillance [5]. Particularly in high income countries, national centres now function as central nodes for national PV efforts and they contribute to building national PV systems by collaborating with other stakeholders be they local, national or international [2, 5, 6].

There is a widespread expectation among several stakeholders including the WHO that national PV systems need to be driven by a national centre [7]. However, previous studies have noted that most national centres in sub-Saharan Africa are currently not the central coordinating bodies of PV efforts in their respective countries [8]. A study by Maigetter et al. [9] revealed that in many countries in Africa, PV functions are not conducted within a separate organization but lumped together with other regulatory functions such as medicines registration, licensing of premises and inspections. The national centre is sometimes a desk in the national medicines regulatory authority (NMRA) with one or two people assigned to carry out all its functions [1, 8, 10]. It is therefore not surprising that the few studies on the features of pharmacovigilance in Africa have arrived at the same conclusion that PV activities performed by African national centres are limited and beset with several challenges of which overcoming a lack of resources is one of the most prominent [1, 11, 12]. This is very different from the situation in developed countries where the national centre is an integral part of the public health system and plays a key role in implementing the national PV agenda [13].

However, there is also evidence that the development of PV systems has become a key priority in certain countries which has led to successes. For instance, the Ghana Food and Drugs Authority has implemented legal provisions mandating Marketing Authorisation Holders (MAHs) to have a Qualified Person for Pharmacovigilance (QPPV) in line with the Public Health Act of Ghana (Act 851, 2012; Part Seven) [14]. The Pharmacy and Poisons Board of Kenya has been designated as a Regional Centre of Regulatory Excellence (RCORE) in pharmacovigilance by the African Union through the African Medicines Regulatory Harmonization (AMRH) programme [15]. Despite this attention, our knowledge on the role and experiences of national centres in Africa is limited especially as it relates to the organizational capacity (resources and relationships) they need to deliver on their mandate. We fill this knowledge gap by providing insight into the activities of national centres that were deemed successful and unsuccessful by the strategic leaders of the centre and by assessing whether the attribution of success or failure is associated with particular types of resources or stakeholders.

## National PV centres and PV initiatives

### National PV centres

The WHO defines a national centre as a single, government-designated centre within a country with the clinical and scientific expertise to collect, collate, analyse and give advice on all aspects related to drug safety [16].The functions [16, 17] of a national centre include, but are not limited to:

- Coordinating of pharmacovigilance activities nationwide;
- Creating awareness on pharmacovigilance among health professionals, healthcare providers, marketing authorization holders and the public;
- Post-marketing surveillance of regulated products;
- Establishing and maintaining a functional national database on ADRs and other medicine related problems to identify unknown or poorly specified adverse effects;
- Leading national and international collaboration on safety issues
- Contributing to the fight against counterfeit medicines

It is obvious from the above that national centres in Africa have a broad mandate and thus require adequate resources to undertake these tasks and to coordinate their national PV systems. The available evidence however suggests that the PV landscape in many African countries is dominated by fragmented PV initiatives and programmes rather than a well-coordinated national PV system [18].

### PV initiatives and programmes

On the African continent, PV activities are often undertaken within public health programmes (PHP) that are executed by the Ministry of Health either alone, or more often, in collaboration with external development partners. Global health initiatives such as the U.S. President’s Emergency Plan for AIDS Relief (PEPFAR) and the US Presidents Malaria Initiative, Global Fund Against HIV/AIDS, TB and Malaria (Global Fund), The Bill and Melinda Gates Foundation’s Malaria Eradication and the adoption of the millennium development goals by the United Nations in the 2000s provided funding for several African countries to combat priority diseases [12, 19, 20]. To qualify to receive this funding, national governments, specifically the Ministries of Health, were tasked to establish formal disease control programmes also known as public health programmes in collaboration with WHO. These programmes were placed under the disease control department of the Ministries of Health and include well known programmes such as the National AIDS/HIV Control Programme, National Tuberculosis Control Programme, National Malaria Control Programmes, the Expanded Programme on Immunization and the lesser known programmes such as the Neglected Tropical Diseases programme. Typically, the programme administrators will draft joint work plans with the development partners providing the funding.

The execution of PHPs resulted in increased access to medicines in African countries but at the same time led to a realisation that safety monitoring systems were largely absent in these countries. This led to calls from the WHO for collaboration among stakeholders to ensure that these countries develop pharmacovigilance systems to protect their populations from medicinal product associated harms [21]. Typically, NMRAs were tasked to collaborate with these PHPs to ensure safety monitoring. As part of this endeavour, several nations in Africa established national centres. The increased funding for PHPs thus was instrumental in the establishment of some national centres in Africa. Most of the established national centres were positioned as individual departments in the NMRA and most still reside within the Ministry of Health [9]. National centres rely on the national government to provide resources for operations, making the national government their most important stakeholder [11]. National centres are also dependent on healthcare professionals, the pharmaceutical industry, academia, PHPs, intergovernmental organizations and development partners who may provide resources to achieve outcomes. Public health programmes rely on spontaneous ADR reporting as the bedrock for collecting safety data on the products used in these programmes and collaborate with the national centres by submitting ADRs directly to the national centres. Sometimes, the national centres also contribute to joint mass drug administration campaigns like deworming of school children with the PHPs through collection and monitoring of ADRs for the safety of patients.

## Methods

This was a qualitative, investigator-administered, semi-structured interview study of strategic leaders in 18 out of 36 national centres in Africa to provide insight into the resource elements, relationships and outcomes they associate with successful and unsuccessful pharmacovigilance experiences.

### Selection

**Fig. 1:**
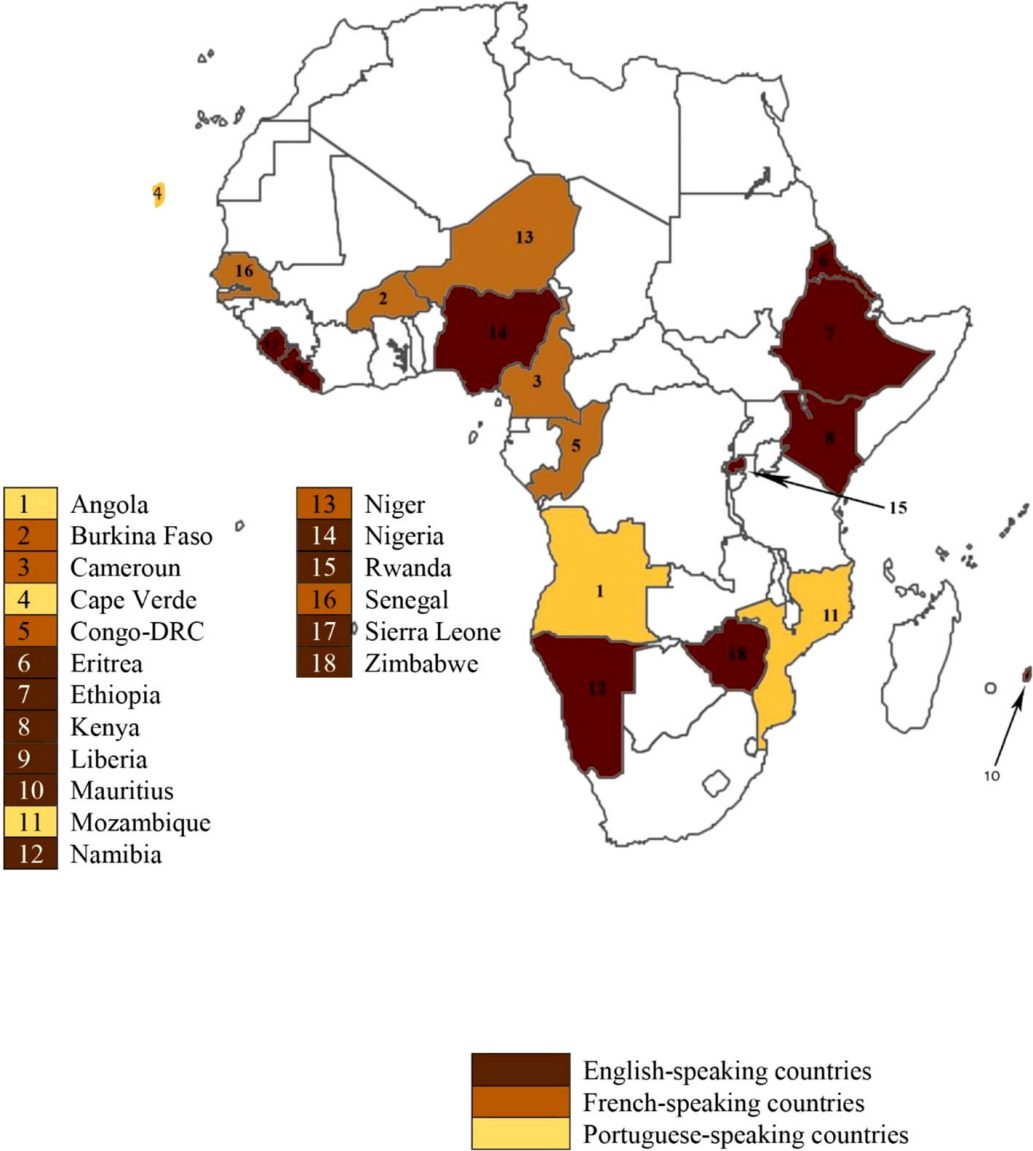
Countries, regions and languages of participants

The participants were purposely selected taking into consideration language (English, French and Portuguese) and region (Central, East, West and Southern Africa) representing sub-Saharan Africa as seen in Fig. 1. To be included in the study, individuals needed to be a current or immediate past employee of a national centre and to be employed in a decision-making role. Sixteen of those interviewed are/were the heads of the national centre in their respective countries.

### Data collection

Interviews were conducted between September 2015 and April 2016. Sixteen interviews were conducted face-to-face and two via phone calls and followed up by emails. The lead investigator had preliminary meetings with participants, explained the research aims and sought verbal consent. Each participant was subsequently interviewed once, with interview duration ranging between 15 and 25 min. The Ghana Health Service Ethics Review Committee’s Standard Operating Procedure, mentions that ethical review is not needed for studies documenting “public behaviour” of professionals working in a public organization [22]. Accordingly, we did not seek ethical approval for this study but conformed with ethical guidance on anonymization of quotes to prevent statements that could be traced back to individuals.

Two pilot interviews led to minor tweaks of the interview protocol and are included in the final data analysis. An interview guide is provided in Additional file 1. In short, participants were asked to describe pharmacovigilance experiences defined as an activity in which the national centre was involved and that had an impact on the delivery of the mandate of the centre as defined in “National PV centres” section. The interviewer asked for three successful and unsuccessful experiences defined as experiences that had a positive or negative impact on mandate delivery, respectively. For each situation the interviewer also asked for reasons why the experience was deemed successful or unsuccessful and asked follow-up questions when needed.

We subsequently analysed these situations to examine how the strategic leaders attributed positive or negative impact to:

- various types of resources (e.g. financial, technical, human, social, political resources) they acquired and how they used them in programme and process management;
- creation and maintenance of relationships with different types of stakeholders (e.g. national government, development partners, intergovernmental organizations, industry, academia, public health programmes)

Thus, a successful experience was defined as national centre relationship with a stakeholder that resulted in the attainment of a resource facilitating the centre to deliver on its mandate. Conversely a negative experience was defined as any national centre relationship with a stakeholder that did not result in the attainment of a resource hindering the centre to deliver on its mandate.

### Coding

Interviews were transcribed verbatim by an experienced co-author (DA). Upon compilation, a total of 18 *3 = 54 successful and 54 unsuccessful experiences were derived. Each experience was subsequently coded for mentioned relationships with stakeholders, mentioned acquired resources and mentioned outcomes. For instance: if a national centre described an experience where they were able to lobby the Ministry of Health/Minister of Health to present a case in Parliament to get a law passed for Marketing Authorization Holders to be held responsible for the safety of their products on the market, the experience was coded as a relationship with the Ministry of Health/Minister of Health, the acquired resource was legal backing and the function was post-marketing surveillance of regulated products. Conversely, a negative experience was defined as any national centre relationship with a stakeholder that did not result in the attainment of a resource. An example is if a national centre was not able to embark on a nationwide training of healthcare workers on ADR reporting because it doesn’t have a budget allocation for such an activity from the national government. The stakeholder mentioned in this case was national government, the resource not provided was financial resources and the function not delivered was creating awareness on pharmacovigilance.

An initial coding of 9 transcribed texts was done manually per participant by the lead investigator (HHA) and reviewed by two authors (JH, AD). For each experience, resources mentioned were assigned to one of 5 resource categories, stakeholders associated with the acquisition of these resources were assigned to one of 6 stakeholder groups and functions fulfilled or not fulfilled were assigned to one of 6 groups. Definitions for each resource and stakeholder groups are provided in Table 1, whereas the six functions of national centres are mentioned in “National PV centres” section. We only considered one dominant resource and stakeholder per experience. In 12 experiences, participants did not mention the stakeholders associated with the resources. Upon completion 108 resources and 96 stakeholders were coded for the combination of successful and unsuccessful experiences. The list of generated codes was compared to the remaining 9 transcribed texts, but no new categories or themes emerged.

**Table 1:** Definitions of resources and relationships used in the study

Type of resource	Definition
Financial resources	Funding or financial capital
Technical resources	Materials and infrastructure (e.g. computers, reporting infrastructure)
Political resources	Law, policy and other legislative instruments
Human resources	Staff and human expertise
Social resources	Relationships including collaborations, partnerships and networks
Type of stakeholder	Definition
National government	The National Regulatory Agency and the Ministry of Health
Development partners	Organizations that work with a variety of in-country partners to improve the lives of poor and vulnerable people in developing countries
Inter-governmental organizations	Organizations comprising mainly of sovereign states
Public health programmes	Organizations responsible for health services to improve and protect community health
Academia	Organizations concerned with the pursuit of education, research and scholarship
Industry	Organizations who market and sell pharmaceutical products

### Analysis

The coded interview data was tabulated using frequency tables*.* Successful and unsuccessful experiences were assessed for frequently mentioned combinations of resources, stakeholders and functions. The combinations of resources, stakeholder and functions that strategic leaders attributed to success or failure were described as themes with verbatim quotes from the participants. National centres in Africa are at varying levels of maturation thus we also compared experiences within country-groupings using the grouping system developed by Management Sciences for Health (MSH) [6]. According to this, Angola, Burkina Faso, Cameroun, Cape Verde, Eritrea, Liberia, Mauritius and Niger are in group 1 - countries with minimal or no capacity for PV. Rwanda, Congo-DRC, Ethiopia, Kenya, Mozambique, Senegal, Sierra Leone and Zimbabwe are in group 2- countries with basic organizational structures. Group 3 countries are countries with the capacity to collect and evaluate safety data based on legal and organizational structure; none of the countries interviewed were in group 3. Namibia and Nigeria are in group 4 - countries that have basic structures for both passive and active surveillance activities. Statistical analysis was not performed.

## Results

Of the 18 participants, there were 8 females and 10 males. Fifteen were pharmacists and 3 were physicians. All the 18 national centres interviewed (except one) were departments under the NMRA.

**Fig. 2:**
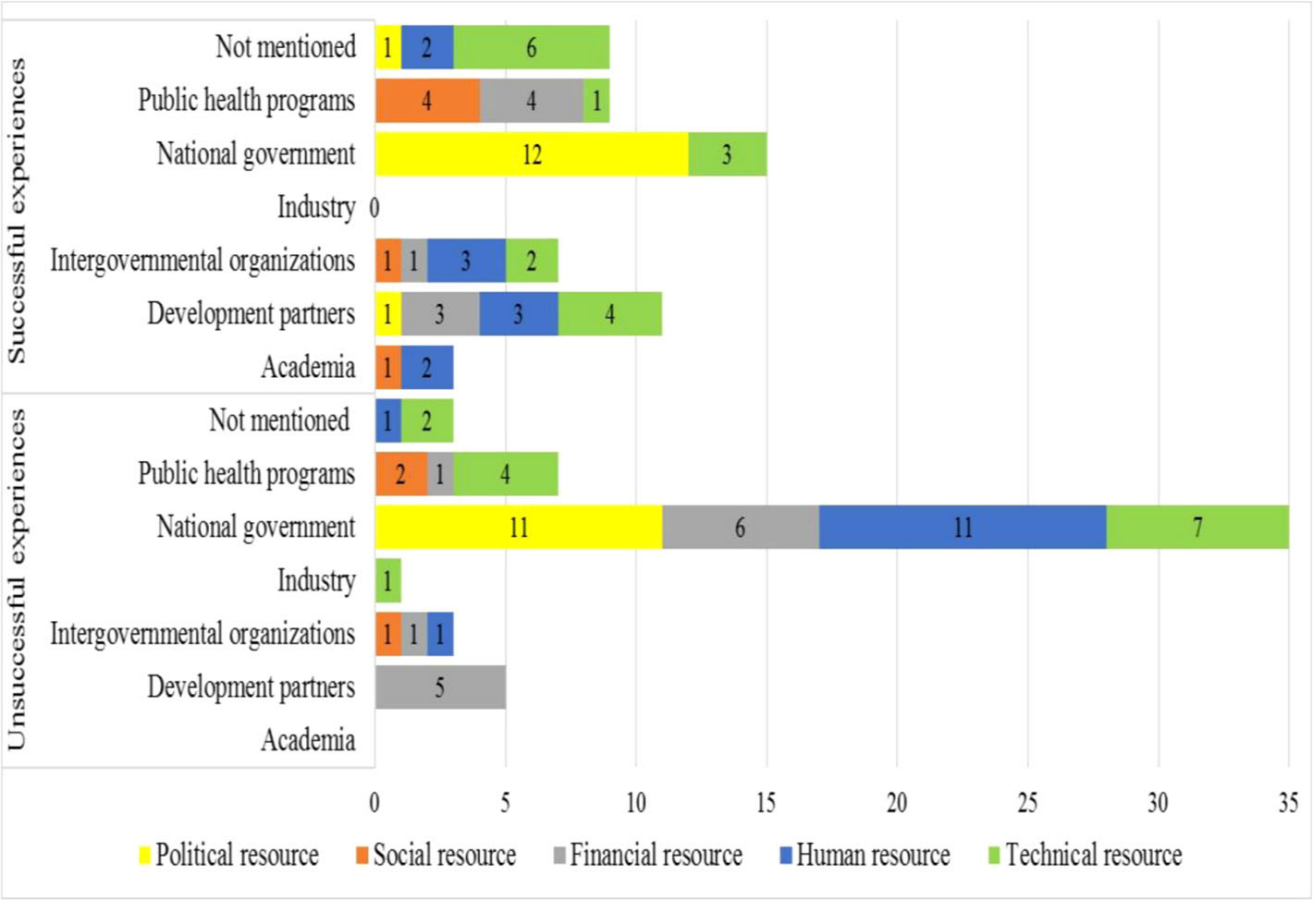
Stakeholders mentioned in the provision of resources

Table 2 provides an overview of the MSH country groupings and the different types of successful and unsuccessful experiences mentioned by participants and the coded resources based on each experience. Figure 2 depicts the dominant stakeholder groups mentioned in association with these resources. Of the 108 experiences collected, participants most often discussed experiences related to the acquisition of technical resources (16/54) such as reporting infrastructure, testing laboratories, phones and vehicles, and political resources (13/54) such as legal mandate, decentralization and political support as successful. Financial resources (15/54) such as grants and dedicated budgets as well as human resources (13/54) such as staffing, capacity building, knowledge were most often described as unsuccessful. Stakeholders that were most often mentioned in experiences by participants were national government (50/108), development partners (16/108) and public health programmes (16/108). The resources and stakeholders associated with these experiences are elaborated on below starting from the most frequently mentioned.

**Table 2:** MSH country groupings, experiences and resources

**MSH Group 1- Countries with minimal or no capacity for PV**				
**Country**	**Successful experiences**	**Successful resources assigned**	**Unsuccessful experiences**	**Unsuccessful resources assigned**
Angola	• Deployment of PV focal persons to various regions of the country, thus decentralizing PV • ADR reports received through positive collaboration with HIV and Malaria Programmes • Funds received through collaboration with development partners	• Political resource • Social resource • Financial resource	• No PV law to enforce regulations • No dedicated budget for PV • No reporting tools	• Political resource • Financial resource • Technical resource
Burkina Faso	• Regulatory framework implemented by government • Deployment of PV focal persons to various regions of the country, thus decentralizing PV • Establishment of national technical committees with tools for PV work	• Political resource • Political resource • Technical resource	• No properly recognized National Regulatory Authority • No dedicated budget for PV • No tools to embark on active monitoring	• Political resource • Financial resource • Technical resource
Cameroon	• Funds received through collaboration with development partners • Continuous receipt of PV literature through established relationship with development partners • PV Decree signed by head of state and minister of health	• Financial resource • Social resource • Political resource	• No dedicated budget for PV • Untrained PV staff • No internet to submit ADR data to VigiFlow	• Financial resource • Human resource • Technical resource
Cape Verde	• Deployment of PV focal persons to various regions of the country, thus decentralizing PV • Improved reporting infrastructure through TV and radio campaigns • Dissemination of ADR data through publication in peer review journals for Portuguese speaking countries	• Political resource • Technical resource • Technical resource	• No PV law to enforce regulations • Inadequate reporting infrastructure • No dedicated budget for PV	• Political resource • Technical resource • Financial resource
Eritrea	• Funds received through collaboration with development partners • Trained PV staff • Deployment of PV focal persons to various regions of the country, thus decentralizing PV	• Financial resource • Human resource • Political resource	• No PV law to mandate reporting by industry • Low AEFI reporting due to poor collaboration with EPI • Pharma industry does not monitor the safety of their products	• Political resource • Technical resource • Political resource
Liberia	• Trained PV staff • Incorporation of PV into curriculum of educational institutions due to effective collaboration with Academia • Availability of tools for active monitoring of drugs from international donors	• Human resource • Social resource • Technical resource	• No dedicated budget for PV • Inadequate human resource for PV activities • No PV law to enforce regulations	• Financial resource • Human resource • Political resource
Mauritius	• Full membership in the PIDM due to positive collaboration with WHO • Improved reporting infrastructure through collaboration with PHPs • Technical support received through collaboration with development partners and PHPs	• Social resource • Technical resource • Social resource	• Inadequate reporting infrastructure • No dedicated budget for PV • No PV law to enforce regulations	• Technical resource • Financial resource • Political resource
Niger	• Deployment of PV focal persons to various regions of the country, thus decentralizing PV • Attending trainings with the Head of the NRA, facilitation of travel by Head of NRA • Tools available to embark on district inspections	• Political resource • Political resource • Technical resource	• Inadequate human resource for PV activities • Untrained PV staff • No dedicated budget for PV	• Human resource • Human resource • Financial resource
**MSH Group 2- Countries with basic organizational structures for PV**				
**Country**	**Successful experiences**	**Successful resources assigned**	**Unsuccessful experiences**	**Unsuccessful resources assigned**
Congo-DRC	• Technical support received through collaboration with development partners and PHPs • Introduction of android smartphones to communicate effectively with health practitioners • More trained human resource from Implementation of Drug Therapeutic Committees(DTC)	• Social resource • Technical resource • Human resource	• Inadequate reporting infrastructure • Untrained PV staff • No dedicated budget for PV	• Technical resource • Human resource • Financial resource
Ethiopia	• Trained PV staff • Introduced PV into national curriculum, to train more human resource for PV • Fulltime MSH employee placed at the national centre to help with PV activities	• Human resource • Human resource • Human resource	• Lack of accredited laboratories • More human resources are needed to deliver on mandate • Poor AEFI reporting infrastructure	• Technical resource • Human resource • Technical resource
Kenya	• Two ministers of state took part in the launch of the PV system. • Launch of online pharmacovigilance electronic reporting system • Funds provided through joint post market surveillance with PHPs	• Political resource • Technical resource • Financial resource	• More human resources are needed to deliver on mandate • Inadequate reporting infrastructure • No PV law to enforce regulations	• Human resource • Technical resource • Political resource
Mozambique	• Deployment of PV focal persons to various regions of the country, thus decentralizing PV • Funds for training received through collaboration with WHO • Availability of legal instruments to promote PV	• Political resource • Financial resource • Political resource	• Untrained PV staff • No dedicated budget for PV • Poor collaboration with PHPs	• Human resource • Financial resource • Social resource
Rwanda	• Trained PV staff • Implemented performance based evaluations for district hospitals • Collaboration with AMRH and EAC-PV harmonization to promote PV activities	• Human resource • Technical resource • Social resource	• Inadequate human resource for PV activities • No dedicated budget for PV • Poor collaboration with PHPs	• Human resource • Financial resource • Social resource
Senegal	• Trained PV staff • Tools available for data analysis and data sharing • Funds for training received through collaboration with NMCP	• Human resource • Technical resource • Financial resource	• No PV staff with data management expertise • No PV representatives in the regions of the country, only the capital region • No dedicated budget for PV	• Human resource • Political resource • Financial resource
Sierra Leone	• Adjustment of malaria treatment due to strong collaboration with NMCP • Deployment of PV focal persons to various regions of the country, thus decentralizing PV • Introduced PV into national curriculum, to train more human resource for PV	• Social resource • Political resource • Human resource	• No dedicated budget for PV • Inadequate reporting infrastructure • No PV law to enforce regulations	• Financial resource • Political resource • Political resource
Zimbabwe	• Donor funding available for PV related projects • Guidance documents and publications available for PV work • AEFI Surveillance systems established since 2001	• Financial resource • Technical resource • Technical resource	• No internet (Wi-Fi) services to submit data to VigiBase • Inability to generate own funds • Inadequate human resource for PV activities	• Technical resource • Financial resource • Human resource
**Group 4- Countries with basic structures for passive and active surveillance**				
**Country**		**Successful resources**		**Unsuccessful resources**
Namibia	• Ministry of Health gave the mandate to setup the national centre • Active surveillance tools available for safety monitoring • Implemented patient reporting system	• Political resource • Technical resource • Technical resource	• Inadequate human resource for PV activities • No dedicated budget for PV • Inadequate spontaneous reporting infrastructure	• Human resource • Financial resource • Technical resource
Nigeria	• Active surveillance tools available for safety monitoring • Funds for training received through collaboration with PHPs • Guidance documents and publications available for PV work	• Technical resource • Financial resource • Technical resource	• No online reporting infrastructure • Inadequate human resource for PV activities • No PV law to enforce regulations	• Technical resource • Human resource • Political resource

Group 1: Countries with minimal or no capacity for PV

Group 2: Countries with basic organizational structures

Group 3: Countries have the capacity to collect and evaluate safety data based on legal and organizational structures

Group 4: Countries that have basic structures for both passive and active surveillance activities

### Experiences involving technical resources

The interviewees mainly made reference to technical resources that facilitated ADR reporting. For instance, participants mentioned that having access to online reporting systems made data readily available and had other benefits.

*“Launching of the online reporting system has helped, it minimizes the paperwork and it is less tedious than the manual reporting”. (Participant 8).*

Reference was also made to technical resources for day-to-day operations. For instance, having vehicles aided post-market surveillance and in mentioning the benefits of acquiring smartphones, a participant mentioned

*“We found that doctors have a problem managing serious ADRs in the field. Our smartphone application allows us (national centre) to communicate with doctors in real time”. (Participant 5).*

In discussing inability to acquire technical resources, lack of data analysis tools, internet, data management infrastructure and accredited laboratories were emphasized.

“*We have only one national laboratory; we are not able to test samples to verify if they are standard or counterfeit when ADRs are reported to us”. (Participant 7).*

#### Stakeholders

Participants expected to acquire basic technical resources such as computers and internet needed for their day to day work from national governments and costly ones from PHPs or development partners.

*“I have ICSRs, but can’t enter into VigiFlow because we don’t have internet connection all the time”. (Participant 3).*

National governments were more often associated with unsuccessful acquisition of technical resources and development partners the most successful acquisition of technical resources.

Participants indicated that they work closely with development partners in their day to day work whether in the provision of tools needed for their work or in the provision of other technical resources.

*“MSH was instrumental in setting up the national centre. They provided technical resources and then later the national centre was incorporated into the structure of the ministry”. (Participant 12).*

National government was lauded for providing space in the national regulatory authority for the national centre and setting up technical committees.

*“The government has set up national commission with tools to validate ADR reports, they have the authority to withdraw or suspend any medicine from the country”. (Participant 2).*

A recurring unsuccessful acquisition of technical resources associated with public health programmes was the inability to deploy mutual surveillance systems between the programmes and the centre to enable efficient data sharing.

“*Vaccine surveillance system is not in place at all at the national centre and the extended programme for immunization, we are currently working on the establishment of such a vaccine surveillance system”. (Participant 6).*

It was mentioned that PHPs sometimes only provided disease-specific resources. For example, a vaccine surveillance system can only fulfil a specific need of a national centre’s mandate and may not be useful for other purposes which leads to national centres having silo surveillance systems as the interviews revealed. Further, it was mentioned that development partners provided technical resources based on their programme objectives. Participants expressed that they tie their work plans to development partners’ agenda even when their needs were different.

*“Working with development partners is sometimes difficult because they decide what level to tie their resources and sometimes the resources are not specific for our needs”. (Participant, 9).*

#### MSH country groupings

It was expected that countries in group 4 would discuss more sophisticated technical resources, however the interviews revealed that countries with different levels of maturation of their PV system discussed similar technical resources. In discussing unsuccessful acquisition of technical resources, two countries in group 4 with basic structures for both passive and active surveillance activities were for instance struggling with online reporting:

*“The issue of reporting online for instance; for some strange reason we haven’t been able to do something as simple as that”. (Participant 14).*

At least one country in each group mentioned successful acquisition of technical resources from development partners. Countries in groups 1 and 2 appear to work more closely with the Global Fund whereas countries in group 4 work with a more varied group of development partners (e.g. John Snow Incorporated (JSI) and United States Pharmacopeia (USP).

### Experiences involving political resources

Political resources such as launching of the pharmacovigilance system by the Minister of Health was used to champion pharmacovigilance to other health professionals and the public. Political support sometimes manifested in the Ministers of Health accompanying national centre personnel on awareness creation campaigns which helped legitimize the national centre as an organization in the healthcare system.

Experiences in which legal mandates were utilized to withdraw harmful products, decentralize PV activities and mandate reporting by industry were also mentioned.

Regarding common successful experiences, three out of the 18 strategic leaders interviewed indicated they had a legal framework or law that specifically mentions pharmacovigilance and a participant described how empowering it can be:

*“The national centre was set up under the NRA with legal framework, guidelines, staff, advisory committee and reporting systems through consultation with all stakeholders”. (Participant, 18).*

Other common successful experiences related to decentralization which seeks to bring pharmacovigilance closer to the patient. Six of the countries interviewed had embarked on decentralization initiatives by establishing regional or zonal centres, sometimes by using Drug Therapeutics Committees (DTCs) in regional public hospitals as was the case in Congo-DRC and Eritrea or by having regional focal persons as was the case in Angola, Cape Verde, Mozambique and Sierra Leone.

*“With the support of the national government, we introduced pharmacovigilance ambassadors in all 4 regions of our country and this has helped increase ICSR reporting”. (Participant 17).*

Unsuccessful experiences when discussing political resources centered on lack of legislations, inability to amend existing Health Bills to include PV and inability to mandate reporting by industry. Five of the countries interviewed had processes in place to implement laws.

*“Pharmacovigilance is not developed in my country because the processes to implement PV law started in 2003 and is ongoing as of 2015”. (Participant 1).*

Participants stated they have had to improvise in the absence of specific PV laws by relying on PV statements in the national regulatory authority laws as legal backing for their work.

*“We have the regulatory authority act which states to ensure safety of products; it sets the pace that this is the intention of government to eventually enact a PV law”. (Participant 14).*

#### Stakeholders

As expected, almost all the political resources were associated with national government. Participants emphasized that only national governments can provide national centres with legitimacy. Successful acquisition of political resources from national government and the accompanying legitimacy was considered an enabling condition which allowed the national centre to mobilise other resources and have stable operations. However, several participants mentioned not having full political backing as an unsuccessful experience. The interviews revealed that in a considerable number of cases, national governments provided initial political resources by enacting policies which aided national centres to become members of the WHO Programme for International Drug Monitoring (PIDM) but failed to continue with this. This also required the national government to launch the national pharmacovigilance system. It is important to note that many successful experiences to do with the acquisition of political resources focus on early stages of the PV system development when legal systems were still being built and new policies being implemented.

*“To start pharmacovigilance, the government adopted two regulatory frameworks; one formed the regulatory authority and the second formed the national centre. These two documents helped start pharmacovigilance activities in the country”. (Participant 2).*

Most participants had challenges with the acquisition of political resources from the national government.

*“In the absence of strong regulatory laws, our country has become a dumping ground of fake products. The current law does not specify pharmacovigilance activities making it difficult to prosecute offenders”. (Participant 9).*

#### MSH country groupings

Countries in group 4 spoke of receiving varied resources from government whilst countries in groups 1 and 2 spoke mainly of political support they have received.

*“I came to this meeting with my Director. She is 2* ^*nd*^ *to the Minister of Health and she facilitated everything”. (Participant 13).*

Irrespective of level of maturation of the PV system, interviewees referred to the absence of specific pharmacovigilance laws when discussing unsuccessful acquisition of political resources. Moreover, none of the countries had autonomous centres. It was unexpected that some countries in group 4 are still working with acts that reference pharmacovigilance and not PV-specific laws.

*“We are not an autonomous agency. The whole idea of our national regulatory agency set up was to remove government bureaucracy so that we can do drug regulation without all those levels of reporting to slow us down”. (Participant 14).*

### Experiences involving financial resources

There were 23 experiences (8 successful, 15 unsuccessful) mentioning financial resources (Table 2). The dominant stakeholder groups associated with financial resources were development partners (8), national government (6) and public health programmes (5).

Most of the national centres interviewed were not income-generating and got their funding from projects and/or from government budgets. Fourteen of the eighteen countries stated they did not have dedicated budget for PV activities.

Successfully attained financial resources were used to acquire other resources, mainly technical and human resource. Participants discussed buying equipment for day-to-day operations (e.g. computers) and sending national centre personnel to international meetings.

Experiences describing lack of financial resources focused mainly on irregularity of funding and lack of autonomy of national centres to generate their own revenue. The interviews revealed that the lack of a stable financial resource stream manifested itself in several ways: firstly, the national centre was not able to undertake key activities such as ICSR collection. Secondly, they are unable to embark on important initiatives such as active monitoring and lastly, national centre personnel are unable to acquire much needed training necessary for their work. Five of the strategic leaders who indicated they were successful in acquiring financial resources also indicated they were unsuccessful in acquiring financial resources usually because some of their efforts didn’t yield results. The inability to generate own revenues was considered particularly problematic when it increased dependency on the government:

*“We are totally dependent on the Ministry; we do not generate our own income hence we are limited in the number of activities we can undertake.”. (Participant 9).*

*“The national centre does not have the autonomy to submit its own budget to the national regulatory authority”. (Participant 16).*

*“I don’t belong to the group who discuss budget, it’s the director (of the NRA), I can propose activities, but the director decides whether we do it or not”. (Participant 3).*

#### Stakeholders

Development partners appeared to play a key role in the provision of financial resources (8/21) but many participants (5/8) mentioned that they are not always able to acquire funding from them. This might be explained by the fact that national centres have typically enjoyed financial resources from development partners which has become part of their resource acquisition strategy.

Some participants elaborated on successful acquisition of financial resources from development partners

*“We receive donor funding for PV projects. 50% of our staff are funded by donor projects”. (Participant 18).*

*“We got financial support from United States Pharmacopeia (USP) and United States Agency for International Development (USAID) to conduct minilabs for malaria and post market surveillance for HIV.” (Participant 8).*

Fear of losing funding, partners not delivering promised funds and funding tied to partners’ goals were some of the concerns expressed by participants in discussing inability to acquire financial resources.

*“Now we are working well with Global Fund but if tomorrow there is no commitment between Global Fund and the country, our activities will be let down. This is a fear I have.” (Participant, 5).*

Discussions on difficulties with acquiring financial resources from national government centred around the unpredictability of funding which hindered planning and forecasting and general inadequate funding to support day to day operations.

*“(Financial) resources are not very predictable. It takes a lot of efforts to have a budget and still the budget is not enough for our priority activities”. (Participant, 4).*

National government was not mentioned in association with the successful acquisition of financial resources because participants had tacit expectations that funding for national centres activities is an action that governments should routinely undertake.

#### MSH country groupings

Participants in groups 1 and 2 discussed the lack of financial resources from national government for basic operations whilst participants in group 4 appear to have stable funding streams.

“*Our funding previously was from donors but now we have funding from government and it is based on our activity plan”. (Participant 12)*.

Participants in groups 1 and 2 discussed acquiring financial resources from PHPs and development partners to embark on awareness creation and training. National governments (6/13) and development partners (5/13) were mentioned most in association with unsuccessful acquisition of financial resources by all groups as seen in Fig. 2.

### Experiences involving human resources

Human resource was mentioned 22 out of 108 times, most often (13/22) in relation to unsuccessful experiences (Table 2). The stakeholder groups mentioned in association with human resource were national government (11/20), intergovernmental organizations (4/20) and development partners (3/20) (Fig. 2).

Successful experiences in acquiring human resources were about using experts from Drug and Therapeutic Committees (DTCs) to do PV work, having regional focal persons and incorporating PV into the curriculum of health disciplines.

Adequate staffing appears to be a challenge for most national centres. In some cases, national centres had to rely on personnel from other departments to offer support in addition to their regular duties (4/13) and, due to competing priorities, PV activities were compromised.

*“I have no time to do PV. In the Direction of Pharmacy (national regulatory authority), we have only 6 personnel for all the work and I have other activities to do”. (Participant 13).*

Moreover, participants emphasized the high personnel turnover at national centres (3/13), such as national centre personnel leaving to go work with development partners, industry and academia because these offer stable work environments.

*“If you train 10 people today, one or two years later only 2 will still be working, the rest disappear to the other organizations”. (Participant 5).*

Politics appears to play an important role in the sustainability of national centre personnel as most strategic positions at the national regulatory authority are occupied by political appointees thus affecting who is nominated as head of the national centre. Whilst participants did not state this explicitly, 3 participants provided strong hints.

*“In Africa most issues are politicized; there have been changes in the system that has weakened the progress we have made in (PV) so far”. (Participant 9).*

#### Stakeholders

National government was associated most with unsuccessful experiences in discussing the inability to acquire human resources (11/13). Participants mentioned challenges such as unavailability of skilled expertise. The interviews revealed that some national centres have collected ICSR data but due to a lack of data analysis expertise have not been able to make decisions out of this data.

*“We use the WHO Method (for causality assessment) but we cannot analyse the data with VigiFlow. We need training”. (Participant, 16).*

National centres are tasked with monitoring the safety of products sold by MAHs. However, the MAH personnel tend be more knowledgeable in PV than national centre personnel. There have been instances where national centres have received documentations from MAHs and have had to rely on the MAHs to explain what the national centre needs to do with such documentation.

*“MAHs sometimes know more about pharmacovigilance than you who is the regulator. It has been a challenge to build the capacity of the national centre staff to regulate the MAHs”. (Participant 14).*

Successes in acquiring human resource were mainly associated with development partners (3 experiences), intergovernmental organizations (3 experiences) and academia (2 experiences). Development partners helped with creation of DTCs, staff augmentation and training.

*“With help from MSH we implemented DTCs in general hospitals to advice the national centre”. (Participant, 5).*

*“We have a full-time MSH staff placed at the national centre. She is supported by MSH”. (Participant 7).*

Intergovernmental organizations were mentioned in relation to capacity-building guidelines and other policy documentations development and human resource benefits from belonging to regional partnerships such as the East African Community (EAC).

*“The EAC harmonization provides us with various expertise from the different countries, for instance we are the lead in Pharmacovigilance whilst other functions such as medicines registration are performed by different countries”. (Participant 8).*

#### MSH country groupings

Lack of adequate human resources both in personnel and expertise was a common theme amongst all three groups. Participants in groups 1 and 2 mentioned not having enough personnel to perform day to day duties whilst participants in group 4 mentioned not having adequate expertise to do active surveillance. Successful acquisition of human resources by groups 1 and 2 were mostly about using the DTCs to augment their operations.

Participants in groups 1 and 2 also mentioned academia as helping augment human resources by incorporating PV into the curriculum of healthcare disciplines.

### Experiences involving social resources

Social resources were mentioned 9 out of 108 times and mainly in association with successful experiences (8/9) (Table 2). The stakeholder groups associated with social resource were public health programmes (6/9), intergovernmental organizations (2/9) and Academia (1/9).

The interviews revealed that national centres constantly seek resources from various stakeholders thus being able to build linkages is key to their survival. Social resources such as collaborations, building partnerships, establishing trust-based relations and networking therefore emerged as a separate theme in successful and unsuccessful experiences.

National centres discussed experiences in which they have been able to build mutually respectful trust-based relationships with some organizations which became instrumental in safety monitoring efforts:

*“Through our strong collaboration with the malaria programme, we embarked on joint monitoring and with the evidence collected we switched our first line of malaria drug from Artesunate+Amodiaquine to Artemether-Lumefantrine*”. (Participant17).

Networking with other national centres were also discussed by some participants as beneficial in exchanging knowledge and best practices. Further, PIDM membership guarantees access to publications and advisory support from the WHO, Uppsala Monitoring Centre and the WHO Collaborating Centres in Ghana and Morocco.

#### Stakeholders

Public health programmes were most often associated with successful acquisition of social resources (4/6) Fig. 2. The interviews revealed that PHPs tend to be well-resourced and use medicines or vaccines in their operations thus making them a key stakeholder to national centres. Some PHPs initiated pharmacovigilance activities in some countries.

*“In 2009 the immunization programme embarked on MenAfriVac vaccination campaign. Our country took advantage of this to start some pharmacovigilance activities”. (Participant 13).*

By virtue of the huge doses of medications administered in public health programmes they tend to be a gold mine for ICSR data.

*“We have good collaborations with malaria, tuberculosis and HIV programmes; majority of our ADRs are from the three programmes. Every quarter we share a report with the programmes, so they can appreciate their contributions”. (Participant, 8).*

Successful acquisition of social resources from academia were about working with the universities to incorporate PV in the curricula of healthcare disciplines.

*“We have developed a framework with the universities to incorporate PV into the teaching of medicine, pharmacy and nursing”. (Participant, 7).*

Finally, a participant indicated that they are encouraged by invitations to conferences and meetings by intergovernmental organizations for the knowledge sharing benefits it produces.

*“I am here in Accra on invitation of WHO-CC attending a conference. If I get copies of these presentations, we will use them to work better when we go back to my country”. (Participant, 3).*

#### MSH country groupings

All three groups discussed the same social resources such as building better relationships with partners, ensuring efficient collaborations and linkages with other national centres. For example, the national centre in Cape Verde (group 1) has taken the lead to get all Portuguese speaking countries in Africa to form a partnership for resource mobilization. As of November 2015, Mozambique (group 2) and Angola (group 1) were on board according to the interviews. Another example is Kenya (group 2) and Rwanda (group 1) who are members of the East African Community harmonization for resource sharing. Countries in groups 1 and 2 appear to hinge their operations on what resources partners can provide.

*“We don’t have funds from the Ministry, sometimes we get support from Global Fund or MSH and it’s not fixed so we are not sure how to plan”. (Participant 1).*

While countries in group 4 did not specifically discuss social resources, they appear to have been able to build long term trust-based relationships with some organizations:

*“MSH is still giving us technical support for active surveillance as we requested from them but not for routine activities”. (Participant 12).*

## Discussion

This paper examined the organizational capacity elements (resources and relationships) that strategic leaders in national centres in Africa typically associate with successful and unsuccessful experiences in order to provide insight into the types of resources and relationships national centres need in order to deliver on their mandate. A key finding is that national centres in Africa appear not to be the central coordinating bodies of PV in their various countries but rather conduct a large part of their activities in project-like settings in close collaboration with public health programmes, development partners, intergovernmental organizations and academia. Moreover, national centres experience difficulties in acquiring different types of resources, particularly from national governments, which has made them reliant on external stakeholders, particularly development partners. The difficulties appear to restrict the abilities of national centres to undertake post-market surveillance of the safety and quality of products marketed in the country and the ability to generate the necessary data for evidence-based decision making.

Resource deficiencies have been previously cited as a barrier to the successful delivery of national centres’ mandate [6, 9, 10, 21, 23]. In a publication in the WHO’s World Medicines Situation series, Pal et al. [8] showed that most national centres in developing countries were severely understaffed and under-resourced with their PV agenda being very much donor-driven. Subsequently, a 2012 assessment of 9 African countries by the USAID-SIAPS programme revealed that regulatory infrastructure for PV is weak with only 41% having a PV national policy, 30% with legislations for ICSR reporting, 28% having legal provisions that required MAHs to report ICSRs and only 17% requiring MAHs to conduct post-marketing surveillance activities. These publications showed that national centres in developing countries have limited organizational capacity. A recent review of pharmacovigilance in resource limited countries (Olson et al. [10]) showed that national centres are still characterized by a lack of capacity to collect data. A study by Ampadu et al. [24] on the features of national centres in Africa showed that with the low numbers of ICSRs reported to VigiBase®® most national centres have insufficient data to provide locally-relevant evidence on the benefits and risks of medicinal products.

Our study goes beyond these studies to distinguish between the various resource elements that centres need to deliver and by associating these resource elements with relevant stakeholders in the PV system. This enables a more nuanced examination of the fundamental requirements for sustainable PV in Africa and the organizational capacity needed by African national centres to deliver on their mandate. Our findings are generalizable in terms of geographic context, language, MSH country groupings and year of joining PIDM (Table 3). There is a bit of over-representation of relatively recently established national centres in group 1–2 systems. Our sampling strategy and the resulting findings are thus particularly pertinent for relatively new centres in the systems with limited capacity for PV. Based on our study, we found 3 core challenges that affect the organizational performance of national centres in Africa.

**Table 3:** National pharmacovigilance centres in Africa (Full PIDM members) [6, 13]

Country	National regulatory authority/national PV centre	Year of joining the PIDM	Included in this study	MSH country group
Angola	Direcao Nacional de Medicamentos e Equipmentos	2013	□	Group 1
Benin	Direction de la Pharmacie et des explorations diagnostics	2011		
Burkina Faso	Direction Générale de la Pharmacie, du Médicament et des Laboratoires	2010		
Cameroon	Direction de la Pharmacie, du Médicament et des Laboratoires	2010	□	
Cape Verde	Agência de Regulação e Supervisão dos Produtos Farmacêuticos e Alimentares	2012	□	
Eritrea	National Medicine and Food Administration	2012	□	
Liberia	Liberia Medicines and Health Products Regulatory Authority	2013	□	
Madagascar	Direction de la Phamacie, des Laboratoires et de la Médecine Traditionnelle	2009		
Mauritius	Pharmacy Board, Ministry of Health and Quality of Life	2014	□	
Niger	Direction de la Pharmacie, des Laboratoires et de la Pharmacopée Traditionnelle	2012	□	
Sudan	National Medicines and Poisons Board	2009		
Swaziland	Pharmaceutical Services Department	2015		
Botswana	Drug Regulatory Services, Ministry of Health and Wellness	2009		Group 2
Congo, Democratic Republic	Direction de la Pharmacie et du Médicament.	2010	□	
Côte d’Ivoire	Direction de la Pharmacie et du Médicament.	2010		
Ethiopia	Food, Medicine and Health Care Administration and Control of Ethiopia	2008	□	
Guinea	Direction Nationale de la Pharmacie et du Laboratoire	2013		
Kenya	Pharmacy and Poisons Board	2010	□	
Mali	Direction de la Pharmacie et des Médicaments	2011		
Mozambique	Departamento Farmacêutico	2005	□	
Rwanda	Department of Pharmaceutical Services	2013	□	
Senegal	Direction de la Pharmacie et du Médicament	2009	□	
Sierra Leone	Pharmacy Board of Sierra Leone	2008	□	
Togo	Direction des Pharmacies, des Laboratoires et des Equipements Technique	2008		
Zambia	Zambia Medicines Regulatory Agency	2010		
Zimbabwe	Medicines Control Agency Zimbabwe	1998	□	
Ghana	Food and Drugs Authority	2001		Group 3
Tanzania, United Republic	Tanzania Food and Drugs Authority	1993		
Namibia	Namibia Medicines Regulatory Council	2009	□	Group 4
Nigeria	National Agency for Food and Drug Administration and Control	2005	□	
South Africa	Medicines Control Council	1992		
Uganda	National Drugs Authority	2008		
Egypt	Egyptian Drug Authority	2002		N/A
Morocco	Direction du Me’dicament et de la Pharmacie	1992		N/A
Tunisia	Direction de la Pharmacie et du Médicament	1993		N/A

N/A: These countries are not included in the MSH groupings

□: Country included in the study

The first challenge is over-reliance on development partners. Pharmacovigilance in most countries started and/or have been facilitated by technical and financial support from development partners, usually the Global Fund, MSH through USAID or the Bill and Melinda Gates Foundation. This has led to a situation whereby national centres align their planning activities with those of the funding partners. Whilst this has been useful in several cases, it has also left national centres vulnerable. Changes of priorities by the development partners have often led to near-cessation of PV activities. Countries are also unable to undertake long-term planning due to uncertainties and volatility of financial support from partners.

The second challenge is the seeming indifference of national governments to provide support after national centres have gained membership of the PIDM. National governments tend to provide some political and modest technical support by designating national centres and launching them publicly. Occasionally, national governments have passed subsidiary legislation to help the work of the national centre. However, in several cases this support seems to evaporate once countries become members of the PIDM leaving national centres bereft of resources. This is reflected in the data published by Ampadu et al. [24] where most national centres in Africa appear to do the barest minimum to gain membership of the PIDM by sending 20 ICSRs to VigiBase®. Thereafter, national centres activities seem to slow down spectacularly with few exceptions. In view of the important role expected by national centres of their governments, it is important for the national centre and other stakeholders to continue advocating to these national governments for long-term resources for their national centres in order to fulfil their expected role of providing the needed safety surveillance infrastructure in their countries.

The third core challenge facing national centres is how to engage all PHPs in a sustainable way. The interview data showed that in nearly all countries, national centres are successful in engaging some but not all PHPs. Establishing trust-based relationships with PHPs require adequate human and technical resources most of which are limited in national centres. Public health programmes are the main providers of data for national centres in Africa [24, 25] hence successful collaboration with them will provide not just the needed data but also associated resources. It is however, difficult to see how this can be done sustainably if national centres rely on these programmes for their resources. Collaboration between national centres and PHPs is accepted as extremely important and beneficial to both organizations and the WHO strongly encourages this as stated in the WHO manual “Pharmacovigilance in Public Health Programmes” [26]. To encourage efficient collaboration with PHPs it would be important to research and provide guidance on the factors underlying successful collaboration between national centres and individual PHPs.

The fight against counterfeit medicines was not mentioned in any of the described experiences. This is surprising given that it is a known and ongoing problem in low and middle-income countries [27, 28]. In an article by WHO, it was estimated that one in 10 medicines in low-income countries are counterfeit and likely responsible for the deaths of tens of thousands of children from diseases such as malaria and pneumonia every year [29]. Several researchers have concluded that to combat this problem regulators will need sustained political will, financial support, tools and technical capacity to enforce quality standards in manufacturing, supply and distribution and a coordinated action from the police, customs officials, and Marketing Authorization Holders [30]. National centres could play a role in this but our analysis did not reveal activities focused on counterfeit medicines as a key priority. To address this problem an effective PV programme with enforcement power is needed. Further, it is also surprising that in only a limited number of experiences industry and academia were mentioned as stakeholders. One of the reasons for this might be that there is little industry and academic activity as pertains to pharmacovigilance in the systems under study.

We provide a number of recommendations based on our findings and discussions. First, to further strengthen and expand PV systems in sub-Saharan Africa it is important to develop approaches that allow for sustainable financial and technical resources for national centres as these resources have been identified by strategic leaders as key impediments to the functioning of national centres. National governments will remain the key expected provider of these resources; however, innovative approaches involving collaboration between development partners, public health programmes, academia and industry could be explored as has also been suggested by Pirmohammed et al. [21]. Such collaborative approaches might also help in preventing a situation where national centres become overly dependent on a single stakeholder. Second, it is important that international organizations like WHO and the Global Fund earmark a certain percentage of funds for medicines and vaccines to be set aside solely for safety surveillance and the maintenance of the safety surveillance and quality infrastructure. Third, mandatory QPPV programmes as required in Ghana and other legally enforceable instruments put responsibility on surveillance and the provision of safety data on the pharmaceutical industry who should be a main provider of safety data to national centres [31]. Finally, academic and research institutions could go beyond incorporating PV in their curricula to embarking on PV research and developing tools and techniques relevant for safety surveillance in their respective national context. They could do this in collaboration with national centres. This will contribute to the development of innovative and pragmatic pharmacovigilance approaches [32] that are highly needed for SSA countries.

## Conclusions

This study concludes that national centres in Africa are faced with 3 core challenges. The first is over-reliance on development partners. The second challenge is the seeming indifference of national governments to provide support after national centres have gained membership of the WHO Programme for International Drug Monitoring (PIDM) and the last core challenge facing national centres in Africa is how to engage all public health programmes in a sustainable way.

## Additional file

Additional file 1: Interview Questionnaire. (DOCX 17 kb)

## Abbreviations

ADR: Adverse drug reaction
AMRH: African Medicines Regulatory Harmonization programme
DTC: Drug Therapeutic Committee
FDA: Food and Drug Authority
HCF: Health Care Facility
HCW: Health Care Worker
ICSR: Individual Case Safety Reports
IPAT: Indicator-based Pharmacovigilance Assessment Tool
JSI: John Snow Incorporated
KNUST: Kwame Nkrumah University of Science and Technology
MAH: Market Authorization Holder
MoH: Ministry of Health
MSH: Management Sciences for Health
PIDM: WHO Programme for International Drug Monitoring
PRS: Patient reporting system
PV: Pharmacovigilance
RCORE: Regional Centre of Regulatory Excellence
SPSS: Statistical Package for the Social Sciences
SSA: Sub-Saharan Africa
USAID: US Agency for International Development
USP: United States Pharmacopeia
WHO: World Health Organization
