# Supplementary material for: Organizational capacities of national pharmacovigilance centres in Africa: assessment of resource elements associated with successful and unsuccessful pharmacovigilance experiences
Source: Global Health. 2018 Nov 16;14:109. doi: 10.1186/s12992-018-0431-0 (PMC6240224; doi:10.1186/s12992-018-0431-0)
Supplement: Supplementary file 1 — Interview Questionnaire. (DOCX 17 kb) [file 12992_2018_431_MOESM1_ESM.docx]

**Additional file 1: Interview Questionnaire**

**Strategic Leaders in National centres in Africa Interview Protocol**

**Topic: Successful and Unsuccessful Pharmacovigilance experiences in Africa**

**Introductory Protocol**

This interview will be in the form of an audio-taped face to face conversation.  For your information, only researchers on this project will be privy to the tapes; which will be destroyed after they are transcribed.  All information will be held confidential. The interview is about 20 minutes.

**Introduction**

I have requested to interview you today because I know you have been active in the field of pharmacovigilance for several years. This study focuses on successful and unsuccessful pharmacovigilance experiences in Africa.  The research does not aim to evaluate your techniques or experiences, nor will the information be used as a tool to penalize you.

Participant (Name, Title, Position and Country): ______________________________________

Interviewer (Name and Title): _____________________________________________________

**Interview Questions**

1.      Describe 3 situations where PV was successful in your country?

*       Why was PV successful?

*       Could this experience be replicated in another African country or is it country specific?

2.       Describe 3 situations where PV was unsuccessful in your country?

*       Why was this activity particularly not successful?

*       Could this experience be replicated in another African country or is it country specific?

3. Is there anything else you will like to discuss with me?
